# Translation and Validation Study of the Hypoglycemia Fear Survey in a Greek Population of Children and Adolescents with Type 1 Diabetes Mellitus and their Parents

**DOI:** 10.3390/children10091458

**Published:** 2023-08-26

**Authors:** Eirini Kostopoulou, Ourania Andreopoulou, Sophia Daskalaki, Eleni Kotanidou, Angeliki Vakka, Assimina Galli-Tsinopoulou, Bessie E. Spiliotis, Linda Gonder-Frederick, Sotirios Fouzas

**Affiliations:** 1 Division of Pediatric Endocrinology and Diabetes, Department of Pediatrics, University of Patras School of Medicine, 26500 Patras, Greece; irekost@upatras.gr (E.K.); aggvakka@outlook.com (A.V.); besspil@gmail.com (B.E.S.); 2 Department of Psychiatry, University of Patras School of Medicine, 26500 Patras, Greece; andreop@upatras.gr; 3 Department of Electrical and Computer Engineering, School of Engineering, University of Patras, 26500 Patras, Greece; sdask@upatras.gr; 4 Unit of Pediatric and Adolescent Diabetes Mellitus, Second Department of Pediatrics, School of Medicine, Faculty of Health Sciences, Aristotle University of Thessaloniki, AHEPA University Hospital, 54636 Thessaloniki, Greece; epkotanidou@gmail.com (E.K.); gallitsin@gmail.com (A.G.-T.); 5 Center for Diabetes Technology, University of Virginia, Charlottesville, VA 22903, USA; lag3g@hscmail.mcc.virginia.edu; 6 Department of Pediatrics, University of Patras School of Medicine, 26500 Patras, Greece

**Keywords:** hypoglycemia, fear of hypoglycemia, HFS validation, type 1 diabetes mellitus (T1DM)

## Abstract

The present study attempted to translate and culturally adapt an established research instrument, the Hypoglycemia Fear Survey (HFS) questionnaire, to the Greek population and evaluate its validity and internal consistency so that it can be used for the assessment of hypoglycemia fear in Greek children and adolescents with T1DM and their parents. One hundred Greek children and adolescents with T1DM, 54 males, 6–18 years old, and one of their parents participated in this validation study. The participants completed the translated Greek HFS, which includes one version for children (CHFS) and one for parents (PHFS). Exploratory Factor Analysis (EFA) was used to assess construct validity. Internal consistency was assessed using Cronbach’s alpha, and convergent validity was established by estimating the correlation coefficients between the scores of the HFS scales/subscales and the different constructs of the Pediatric Quality of Life Inventory. The CHFS and PHFS exhibited adequate internal consistency for the total score and the Worry subscale, but lower consistency for the Behavior subscale. High test–retest reliability was also shown. We conclude that the Greek version of the HFS is a valid and reliable instrument to assess the fear of hypoglycemia in Greek children and adolescents with T1DM and their parents.

## 1. Introduction

Type 1 diabetes mellitus is characterized by the body’s inability to regulate blood glucose (BG) levels due to autoimmune destruction of the insulin-producing pancreatic β-cells. Despite the progress that has been made in recent years to treat diabetes optimally, hypoglycemia remains one of the most acute complications of the disease. Hypoglycemia is defined as “any abnormally low plasma glucose concentration that exposes the subject to potential harm”, and the proposed threshold for the plasma glucose value is 70 mg/dL [1].

Hypoglycemia is caused by a mismatch between the BG levels and the circulating insulin concentrations, or by an increased sensitivity to insulin. Symptoms related to hypoglycemia include tachycardia, tremor, sweating, hunger, irritability, dizziness, and confusion. In its more severe form, it may result in seizures, coma, or even death [2]. Treatment for hypoglycemia typically involves consuming some type of fast-acting carbohydrate to raise the glucose level.

Knowing the potential major consequences of hypoglycemic episodes may lead individuals with T1DM or their caregivers to take action in order to achieve adequate glucose control. In some cases, however, overreaction due to excessive anxiety may negatively affect self-management behaviors or proper actions for glycemic control and eventually lead to a mental state called fear of hypoglycemia (FoH) [3,4]. More specifically, this phobic fear of low BG may lead children with T1DM or their parents to engage in behaviors that purposefully maintain high BG levels. Such behaviors include excessive BG monitoring, frequent awakening during the night to check the BG, consumption of unnecessary bedtime snacks, administering less than recommended amounts of insulin, and the avoidance of physical activity [5].

The most well-established research tool for assessing the FoH in children and adolescents with T1DM and their parents is the pediatric Hypoglycemia Fear Survey (HFS), which includes two versions, one for children’s reports (CHFS) and one for parents’ reports (PHFS) [6,7]. The pediatric HFS is a 25-item self-report questionnaire, which comprises two subscales, the Behavior and the Worry subscales. The Behavior subscale was designed to trace any “wrong” or inappropriate self-management behavior systematically followed by children or their parents with the intention to avoid hypoglycemia. In contrast, the Worry subscale was designed to measure excessive concerns regarding the negative consequences of hypoglycemia. The Behavior subscale includes 10 items (e.g., keeping BG higher when I am going to be alone, reducing the dose of insulin whenever I think my BG may fall, etc.), whereas the Worry subscale includes 15 items (e.g., worrying about not having food or drink available when BG is falling, worrying about feeling dizzy or fainting in public due to low BG, etc.). All items are scored on a 5-point Likert scale ranging from never (0) to almost always (4); therefore, higher scores reflect greater fear of hypoglycemia. A total CHFS score is obtained by adding the Behavior and Worry subscale items.

Previous studies have demonstrated the reliability and validity of the English versions of the pediatric HFS [6,8]. Also, the psychometric properties and construct structures of the English, Norwegian, and Italian versions of the HFS have been studied [9,10,11]. One additional study investigated the psychometric properties of the HFS-PYC, an HFS variant designed specifically for parents of young children aged 2 to 8 years [12]. 

The aims of our study were (1) to translate and culturally adapt the HFS questionnaires (i.e., parent’s and children’s versions) to the Greek population, and (2) to evaluate their validity and internal consistency, so that they can potentially be used in the future as research tools for the assessment of hypoglycemia fear in Greek children and adolescents with T1DM and their parents. In order to strengthen the final outcome of this effort, we compared the results of our factor analysis with those of previous studies performed in different countries and in different languages.

## 2. Materials and Methods

### 2.1. Participants

A total of 100 Greek children and adolescents with T1DM, 54 males and 46 females, aged 6–18 years old (mean ± sd: 13.7 ± 3.3), participated in this validation study. The participants were all being followed at the outpatient clinics of the pediatric endocrine departments of two medical centers in Greece, the University General Hospital of Patras in Southern Greece and the AHEPA General Hospital of Thessaloniki in Northern Greece. The age at onset of T1DM of the participants varied from 1.25 to 16.50 years old (mean ± sd: 8.6 ± 3.9), the duration of diabetes varied from 3 months to 16.73 years (mean ± sd: 5.1 ± 4.1), and current HbA1c concentrations varied from 5.0 to 9.4% (mean ± sd: 7.3 ± 1.0).

For each child or adolescent, one of the parents involved with the patient’s diabetes care was also included in the study. The study was conducted during their routine medical appointments in one of the two hospitals, and the participants were asked by their physicians to complete the Greek version of the Hypoglycemia Fear Survey (HFS). Criteria for exclusion were either significant comorbidities in the children that could affect their psychosocial status, quality of life, and FoH, or cognitive and learning disabilities that would preclude their ability to complete the study protocol. In addition to the HFS, participants (both children and parents) were asked to complete the Greek child and parent self-report version of PedsQL™ 3.0 Diabetes Module, which is designed to measure the health-related quality of life (HRQOL) in children with diabetes aged 6–18 years. For the younger children (6–7 years old) who had not yet learned to read well, the questions were asked by the physician. Lastly, sixteen of the participating children and their parents agreed to re-fill the HFS questionnaire three weeks after the first completion, so that the test–retest reliability could be assessed.

The study was approved by the Research Ethics Committee of the University Hospital of Patras (IRB number: 348/9.5.2017) and is in full accordance with the Declaration of Helsinki of 1975, as revised in 2013. Written informed consent was obtained from the parents and informed assent from the participating children and adolescents.

### 2.2. Translation and Validation Procedure

Permission for this translation and validation study was obtained from our co-author Dr. Linda Gonder-Frederick, the original author and creator of the scale. In order to proceed with its translation and adaptation, we followed the nine-step procedure recommended for cross-cultural adaptation of patient-reported outcomes research by Wild [13]. Specifically, two forward translations were produced from the original language (source language) to the target language. Bilingual translators (EK and OA), whose mother tongue is the target language, produced the two independent translations. The two translators had different academic profiles, and each produced a written report. The translations were then compared and discrepancies between them were resolved through interactive discussions between the translators. This last step converged into a single translation. Finally, a third translator (BES), who is an expert in the field of Pediatric Endocrinology, a native English speaker, and blind to the original English version, back-translated the instrument to the source language to ensure that the translation reflected the same item content as the original version. 

Content and face validity were tested using cognitive interviews with 20 children and one parent for each one of them. No negative comments or remarks about confusing content or wording of the items in the scale were made. All items were reported as relevant and clear by the participants. The final back translation was reviewed and approved by Dr. L. Gonder-Frederick.

### 2.3. Greek HFS Questionnaire

The Greek HFS questionnaire as translated and studied in the present study consists of the children’s version (CHFS) and the parents’ version (PHFS). The CHFS includes 25 items and the PHFS 26 items. There is a one-to-one correspondence between the items of the CHFS and the PHFS, with the exception of the 11th PHFS item in the Behavior subscale (“I check my child several times in the middle of the night”), which is not included in the CHFS version. Therefore, the Behavior subscale of the PHFS comprises 11 items and the Worry subscale 15 items, all scored on the same 5-point Likert scale as the CHFS. Until a very recent study by Tumini et al. (2022), which also studied the 26-item Italian version of the PHFS, all previous studies on the PHFS referred to a scale of 25 items [11].

### 2.4. Statistical Analysis

The participants’ responses were initially cross-checked and cleansed of possible errors and misalignments, and missing values were recorded. For all items of the scales, the mean values and standard deviations were calculated from all corresponding participants. *p*-values < 0.05 were considered statistically significant.

The construct validity and internal consistency of the two translated scales were evaluated using Exploratory Factor Analysis (EFA), since this was the first attempt to explore and validate the factor structure of the scales translated into Greek. Following the recommended procedure for EFA [14], the correlations for all item pairs were calculated first. Only items that correlated fairly well, albeit not perfectly, with some other items could be considered further for the analysis. In the present study, the value 0.33 was used as the threshold for item exclusion. The suitability of the data for factor analysis was tested using the Kaiser–Meyer–Olkin (KMO) statistic, for which the bare minimum of 0.5 is recommended [15]. The strength of the relationship among the items was assessed through Bartlett’s test of sphericity, which was significant, as required for the analysis to be completed.

For factor extraction, extensive experimentation with different factor extraction techniques and rotation methods was required. The number of factors was determined following the three methods known to be used for this decision-making process: (1) the Kaiser criterion (retain factors with eigenvalues greater than 1), (2) the scree plot (retain the number of factors indicated by the point where the scree plot levels off), and (3) the Parallel Analysis (run a Monte Carlo simulation and retain those factors for which the eigenvalue attained by the FA is larger than the value obtained from the simulation) [14,15,16]. The extraction method that gave the best results was Principal Axis Factoring (PAF) with promax rotation, in which factors are allowed to be oblique (i.e., non-orthogonal) because they may be correlated pairwise. 

To assess the reliability of the HFS scales, their internal consistency was measured using Cronbach’s alpha separately for each factor revealed. In addition, in order to assess the convergent validity of the HFS, the participants also completed the Pediatric Quality of Life Inventory (PedsQLTM 3.0 Diabetes Module), which is a widely used instrument designed to measure the health-related quality of life (HRQOL) in families with children aged 2–18 years old diagnosed with T1DM [17]. Convergent validity was assessed by estimating the correlation coefficients between the scores of the HFS scales/subscales/factors and the different subscales of the PedsQL™ 3.0 Diabetes Module. 

Lastly, to test for temporal stability in the participants’ responses, the test–retest reliability was assessed. Sixteen children and their parents agreed to re-fill the HFS questionnaire 3 weeks after the first completion, and test–retest reliability was assessed by calculating the Pearson correlation coefficients for the HFS total scale scores, Behavior, and Worry subscale scores. All statistical analyses described above were executed using SPSS 27 (IBM Corp, Armonk, NY, USA).

## 3. Results

### 3.1. Item Exploration and Descriptives

Of 100 parents, 84 responded to all items, 12 missed one item, 3 missed two or three items, and only 1 missed six items from the Behavior subscale. For the children, there were fewer missing values: 89 out of 100 responded to all 25 items, 10 missed one item either from the Behavior or Worry subscale, and only 1 missed two items from the Worry subscale. 

The mean value and the standard deviation of each item of the Greek PHFS and CHFS are presented in Table 1, which displays the approved back-translation questions of the PHFS and the CHFS. The mean item values range from 0.43 (W7) to 3.55 (B8) for the PHFS and from 0.31 (W5) to 3.27 (B8) for the CHFS. Parents and children achieved their highest mean value on the same item (B8). There was also agreement on the items with the three lowest mean values (W5, W7, and W11).

### 3.2. HFS Construct Validity

The correlation matrix for the PHFS items indicated that B1, B2, B5, B6, B8, B9, B10, and B11 had low correlation, less than 0.33, with all other items. Therefore, these items were eliminated from further consideration. The 18 remaining items correlated more strongly with other items and were eligible for the analysis. The reduced dataset gave a KMO statistic equal to 0.891, which, according to Kaiser’s taxonomy, indicates great sampling adequacy, and a significant Bartlett’s test of sphericity (*p* < 0.001), further emphasizing the adequacy of data for factor analysis. 

The factor extraction process included a three-factor model that loads the remaining 3 items from the Behavior scale (B3, B4, and B7) to one factor and splits the 15 items from the Worry scale into two factors (Table 2). The items load with high loadings onto the three factors, all above 0.47 and up to 0.88, explaining approximately 60% of the total variance. The first factor (B3, B4, and B7) measures the tendency of parents to keep BG higher than normal because of FoH and is named “Actions for Maintaining High BG”. The second factor (W1–W4, W6, W9, W10, W12–W15) measures the anxiety parents exhibit related to the child not having help when hypoglycemia occurs or to negative physical consequences due to hypoglycemia and is named “Helplessness/Worry About Low BG”. The third factor (W5, W7, W8, W11) measures the excessive anxiety parents exhibit due to the possible consequences low BG may have on their children’s social image and relationships and is named “Worry About Negative Social Consequences”. Furthermore, the internal consistency of all three factors was quite high (0.85, 0.93, and 0.79, respectively), as measured by Cronbach’s alpha.

For the CHFS, all 25 items were initially included in the analysis since they all correlated well with some of the other items. However, when the factor extraction techniques were applied, B6 exhibited very low communality, i.e., it did not share a large percentage of its variance, so it was dropped. The factor model with the remaining 24 items gave 0.747 for the KMO statistic, which is classified as good, and a significant Bartlett’s test (*p* < 0.001). The factor extraction process for the CHFS led to a four-factor model explaining approximately 41% of the variance. Each subscale comprises two factors (Table 3) with item loadings that vary from 0.34 to 0.82. The items B1, B3, B4, and B7 are loaded to the first factor of Behavior and they all refer to “Actions for Maintaining High BG”, while B2, B5, and B8-B10 are loaded to the second factor and refer to “Actions for Avoiding/Preventing Low BG”. The items of the Worry subscale also split into two factors: W4, W6, W9, and W13–W15 measure the excessive anxiety caused by the FoH (Helplessness/Worry About Low BG), and W1–W3, W5, W7, W8, and W10–W12 measure the excessive anxiety caused by possible consequences of low BG, especially while in public (Worry About Negative Social Consequences). Using Cronbach’s alpha, the internal consistency of each factor was measured at 0.67, 0.73, 0.73, and 0.85, respectively.

Since the factors extracted for the HFS scales were allowed to be oblique, the analysis additionally provided the correlation coefficients between the factors of each scale (Table 4 and Table 5). Strong positive correlations were measured between the two Worry factors in both scales (0.663 and 0.626, respectively), and moderate positive correlations were measured between the two CHFS Behavior factors (0.402). All the other pairs were very weakly correlated.

### 3.3. Convergent Validity

The pairwise correlations between factors of the HFS and the subscales of the PedsQLTM 3.0 Diabetes module are presented in Table 6. Peds1-11 (problems with diabetes symptoms) and Peds23-25 (worries regarding disease-related complications) exhibit the highest positive correlation with the Worry subscales of both the PHFS and the CHFS. Peds12-15 (barriers with the treatment) and Peds16-22 (problems of adherence to the treatment) correlate significantly with the Worry subscales of the CHFS. The Behavior subscale factors do not correlate significantly with any of the PedsQL factors, with the exception of one CHFS Behavior factor that correlates significantly with PedsQL Total.

### 3.4. Test–Retest Reliability

The correlation coefficients between the Behavior and Worry subscales and total scale scores for the sixteen children and their parents who agreed to participate in the test–retest reliability study were as follows: 0.475 (*p* = 0.063) for the PHFS Behavior subscale, 0.752 (*p* < 0.001) for the PHFS Worry subscale, 0.745 (*p* < 0.001) for the PHFS total scale, 0.811 (*p* < 0.001) for the CHFS Behavior subscale, 0.794 (*p* < 0.001) for the CHFS Worry subscale, and 0.878 (*p* < 0.001) for the CHFS total scale. Correlation is significant at the 0.05 level (2-tailed).

## 4. Discussion

The present study describes the translation of the pediatric HFS questionnaire in Greek and the subsequent validation process using a study group of Greek children and adolescents with T1DM and their parents. The pediatric HFS questionnaire, issued separately for children and adolescents with T1DM (CHFS) and their parents (PHFS), has been translated into several different languages [10,11], and multiple studies present results on its validity, internal consistency, and psychometric properties [8,9,10,11,12]. 

Our results on the construct validity of the Greek HFS are in agreement with these pre-existing studies. The application of factor analysis to our data suggested three factors for the PHFS and four factors for the CHFS, similar to previous studies [9,10]. 

Summarizing the EFA findings of the current study, the items that were designed to measure actions for avoiding low BG, such as B2 (“I avoid leaving my child when his/her blood sugar may be low”), B5 (“I immediately give my child something to eat with the first symptom of hypoglycemia”), B11 (“I wake up in the middle of the night in order to check if my child is okay or to check my child’s blood sugar”), etc., were possibly mistaken by the parents as “appropriate” actions for taking care of their child and not as “inappropriate” behaviors related to fear. The elimination of these items from our analysis is in line with previous studies, raising questions about the validity of the original PHFS Behavior subscale [6,10], since in its present version, it measures a mixture of actions that are not all related to fear. According to our results, only those parental actions which intend to maintain the BG of their child higher than medically recommended are in fact related to FoH and should be measured using the Behavior subscale. This is further supported by the high value of the internal consistency of the factor “maintain high BG” (0.85). On the other hand, in the CHFS Behavior subscale, the actions for maintaining high BG form one factor and those preventing low BG form another factor. Both factors contribute to measuring the FoH, as their internal consistencies are acceptable, albeit lower than that of the PHFS (0.67 and 0.73, respectively). In other words, the FoH in parents manifests as behaviors that intend to maintain their child’s BG higher than recommended, whereas in children, it manifests either as behaviors that intend to maintain the BG high or as behaviors that prevent low BG.

In addition, the present study showed that the Worry subscales of both the PHFS and the CHFS provide more solid and direct measures of the FoH, which has also been demonstrated in previous studies [6,10]. EFA revealed two factors for the Worry items of both questionnaires, one measuring the feeling of helplessness and the worry about low BG and the other measuring the worry about possible negative social consequences of low BG. Also, the internal consistency of each factor was high for both the PHFS and the CHFS (Table 2 and Table 3). While many studies on the HFS include these two factors on the Worry subscale, the items allocated to these factors may be different in the parents’ scale and the children’s scale or in different populations, which may reflect major cultural differences and/or different perceptions of what negative social consequences are. Similarly, depending on the study group, certain items may be allocated to either of the two Worry factors, which can be explained by the significant positive correlations found between these factors. A positive but weaker correlation has also been found between the two CHFS Behavior factors. Furthermore, the existence of significant pairwise correlations between the Worry factors and between the Behavior factors suggests an overlap between the involved items, which explains why these factors do not form separate subscales of the HFS.

Another important finding of the present study is that the HFS questionnaire is a highly reliable instrument for use in children and adolescents and their parents, as indicated by the calculated measures of internal consistency. Specifically, the Cronbach’s alphas calculated for the CHFS and the PHFS factors suggest adequate to excellent internal consistency for both factors of the Worry subscale, and adequate or excellent internal consistency for the Behavior factors of the CHFS and the PHFS, respectively. 

The analysis for convergent validity of the Greek HFS questionnaire was the next step in our study and the Greek version of the PedsQLTM 3.0 Diabetes Module was used for this purpose. The PedsQLTM 3.0 Diabetes Module includes 28 items split into five subscales: (1) disease-related symptoms (e.g., feeling hungry, having stomach aches, feeling tired, etc.) (11 items), (2) possible treatment barriers (4 items), (3) degree of adherence to the treatment (7 items), (4) worry regarding disease-related future complications (3 items), (5) difficulties the patient may have in communicating his/her problem to others (3 items). For each question, the 5-point Likert scale was used to rate the responses, ranging from “Never faced the problem” (0) to “I face the problem almost always” (4). Therefore, higher scores indicate more problems associated with the disease and its treatment, more worries, and eventually, worse quality of life. The pairwise correlations between the HFS factors and the five subscales of the PedsQLTM 3.0 (Table 5) suggest that there is an association between the FoH and lower quality of life for the patients and their families [18], which is in line with previous studies [11]. Furthermore, our study suggests that the hypoglycemia-related worries identified in children and adolescents with T1DM are positively associated with disease-related symptoms, treatment problems, and potential future complications. 

Other evidence from this study supports the consistency of the instrument over time, as demonstrated by its high test–retest reliability in a 3-week interval. All corresponding pairs of subscales and total scores correlated strongly, except for the PHFS Behavior subscale, which exhibited moderate correlation. Nevertheless, it is worth noting that test–retest agreement is not always expected in psychometric traits, since anxiety, worrying, and fear levels may vary with time. 

Limitations of the study include the relatively small sample size. Also, although the sample was drawn from two different centers, one from Northern and one from Southern Greece, it may not be representative of the entire country.

## 5. Conclusions

In conclusion, the present study offers, for the first time, data that confirm the usefulness of the Greek version of the HFS questionnaire as an acceptable research tool for assessing the FoH in Greek children and adolescents with T1DM in a clinical setting. This is in agreement with previous research findings from other countries. The results of our analysis support the validity of the HFS, with the Worry subscale being more consistent than the Behavior subscale, as well as the reliability of the instrument. As a result, we conclude that this valid and reliable tool for assessing the fear of hypoglycemia, albeit not optimal, can be used to guide conversations about fear between patients with T1DM or their caregivers and medical personnel. The ability to assess the fear of hypoglycemia in children and adolescents with T1DM and their parents is of major importance, as measures of support can be initiated by healthcare providers. In the case of the parents, these measures are important also in order to prevent the influence this fear may have on children’s psychology, diabetes management, and long-term complications. It is also noteworthy that Behavior items that are excluded by the statistical analysis as measures of the FoH may still be important, as they provide useful clinical information. Therefore, additional research and international collaboration between researchers is needed in order to further improve the scale and the interpretation of the results.

## Figures and Tables

**Table 1:** Back-translated items of the Greek HFS.

Items	Mean (SD)
PHFS	
Behavior Subscale	
B1. I give my child a large bedtime snack	0.68 (0.89)
B2. I avoid leaving my child by himself/herself when his/her blood sugar may be low	2.52 (1.64)
B3. I allow my child’s blood sugar to be a bit higher for greater safety	1.42 (1.05)
B4. I maintain my child’s blood sugar higher when alone for a while	0.95 (1.04)
B5. I immediately give my child something to eat whenever he/she has the first symptom of hypoglycemia	3.20 (1.28)
B6. I decrease my child’s insulin dose whenever I think that his/her blood sugar is very low	1.54 (1.40)
B7. I maintain my child’s blood sugar higher whenever he/she is going to be away from me for a while	1.41 (1.25)
B8. I give my child fast-acting sugar to have with him/her	3.55 (1.06)
B9. I restrict my child’s activity whenever I think that he/she may have low blood sugar	1.99 (1.48)
B10. I check my child’s blood sugar often whenever he/she is going to go out	3.32 (1.08)
B11. I wake up in the middle of the night to check if my child is okay or to check my child’s blood sugar	2.93 (1.41)
Worry Subscale	
W1. My child won’t recognize or realize that he/she has hypoglycemia	2.08 (1.65)
W2. My child won’t have food, fruit, or juice with him/her	1.98 (1.76)
W3. My child may feel dizzy or faint in public	1.74 (1.74)
W4. My child may become hypoglycemic while he/she is sleeping	2.37 (1.53)
W5. My child or his/her friends may be embarrassed at a social occasion	0.57 (1.03)
W6. My child may have hypoglycemia while he/she is alone	1.99 (1.59)
W7. My child may look stupid or clumsy in front of others	0.43 (1.05)
W8. My child may lose control of his/her behavior due to low blood sugar	0.79 (1.19)
W9. No one will be around to help my child when he/she is hypoglycemic	1.87 (1.65)
W10. My child may make a mistake or have an accident at school	1.50 (1.55)
W11. My child may get bad grades at school because something happens when his/her blood sugar is low	0.51 (0.93)
W12. My child may have seizures	1.02 (1.54)
W13. My child may have long-term complications due to frequent hypoglycemia	1.60 (1.62)
W14. My child may feel dizzy or faint when his/her blood sugar is low	1.76 (1.51)
W15. My child may have a hypoglycemia	2.44 (1.30)
Behavior Subscale	
B1. I eat a large snack before I go to sleep	0.83 (0.92)
B2. I try to avoid being alone when my blood sugar may be low	1.77 (1.50)
B3. I maintain my blood sugar high in order to be safe	1.17 (1.25)
B4. I maintain my blood sugar higher whenever I am going to be alone	0.78 (1.11)
B5. I eat something as soon as I have the first sign of low blood sugar	2.50 (1.55)
B6. I reduce my dose of insulin whenever I think that my blood sugar may fall	1.77 (1.44)
B7. I maintain my blood sugar higher whenever I am not at home	1.15 (1.21)
B8. I always carry drinks or food with sugar	3.27 (1.29)
B9. I try not to exercise very much whenever I think that my blood sugar may be low	2.30 (1.56)
B10. I check my blood sugar very often whenever I am not at home	2.44 (1.34)
Worry Subscale	
W1. That I won’t be able to recognize that I have low blood sugar	1.05 (1.15)
W2. That I won’t have food, fruit, or juice with me when my blood sugar is falling	1.16 (1.44)
W3. That I may become dizzy or faint in public due to low blood sugar	0.91 (1.38)
W4. That I may have low blood sugar while I am sleeping	1.41 (1.19)
W5. That I may be put in a difficult position due to low blood sugar	0.31 (0.80)
W6. That I may have a low blood sugar episode while I am alone	1.21 (1.12)
W7. That I may look stupid or clumsy in front of others	0.37 (0.86)
W8. That I may lose control due to low blood sugar	0.80 (1.02)
W9. That there will be no one around when I have low blood sugar	0.90 (1.18)
W10. That I may make a mistake or have an accident at school	0.83 (1.09)
W11. That I may get in trouble at school because something happens when my blood sugar is low	0.37 (0.76)
W12. That I may have seizures	0.43 (0.84)
W13. That I may have long-term complications due to low blood sugar	0.62 (1.00)
W14. That I may feel dizzy or confused when my blood sugar is low	1.21 (1.23)
W15. That I may have low blood sugar	1.69 (1.10)

**Table 2:** Extracted factors for the PHFS scale and item loadings.

Factors	Items	Loadings
Maintaining High BG	B3. I allow my child’s blood sugar to be a bit higher for greater safety	0.71
Cronbach’s *a* = 0.85	B4. I maintain my child’s blood sugar higher when alone for a while	0.86
	B7. I maintain my child’s blood sugar higher whenever he/she is going to be away from me for a while	0.85
Helplessness/Worry About Low BG	W1. My child may not recognize or realize that he/she has hypoglycemia	0.55
	W2. My child may not have food, fruit, or juice with him/her	0.47
Cronbach’s *a* = 0.93	W3. My child will feel dizzy or faint in public	0.88
	W4. My child will become hypoglycemic while he/she is sleeping	0.80
	W6. My child will have hypoglycemia while he/she is alone	0.74
	W9. No one will be around to help my child when he/she is	0.86
	W10. My child makes a mistake or has an accident at school	0.71
	W12. My child may have seizures	0.62
	W13. My child may have long-term complications due to frequent hypoglycemia	0.66
	W14. My child may feel dizzy or faint when his/her blood sugar is low	0.87
	W15. My child may have a hypoglycemic episode	0.82
Worry About Negative Social Consequences	W5. My child or his/her friends may be embarrassed at a social occasion	0.79
	W7. My child may look stupid or clumsy in front of others	0.87
Cronbach’s *a* = 0.79	W8. My child may lose control of his/her behavior due to low blood sugar	0.69
	W11. My child may get bad grades at school because something happens when his/her blood sugar is low	0.59

**Table 3:** Extracted factors for the CHFS scale and item loadings.

Factors	Items	Loadings
Maintaining High BG	B1. Eat large snacks just before bedtime	0.53
Cronbach’s *a* = 0.67	B3. Keep a higher BG to be on the safe side	0.44
	B4. Keep BG higher when alone	0.59
	B7. Keep BG higher when I plan to be away from home	0.78
Avoid/Prevent Low BG	B2. Try not to be alone when BG might be low	0.56
Cronbach’s *a* = 0.73	B5. Eat something right at the first symptom of low BG	0.53
	B8. Always carry some food or drink with sugar	0.61
	B9. Avoid exercise when I think BG might be low	0.53
	B10. Check my BG often when I am away from home	0.66
Helplessness/Worry About Low BG	W4. Having hypoglycemia while asleep	0.62
	W6. Having hypoglycemia when alone	0.39
Cronbach’s *a* = 0.73	W9. To be no one around me during hypoglycemia	0.35
	W13. Getting long-term complications due to low BG	0.34
	W14. Feeling dizzy or fainting due to low BG	0.57
	W15. Having low BG	0.77
Worry About Negative Social Consequences	W1. Not recognizing my BG being low	0.36
	W2. Not having food, fruit, or juice with me when BG is dropping	0.54
Cronbach’s *a* = 0.85	W3. Feeling dizzy or faint in public because of low BG	0.82
	W5. Embarrassment due to low BG	0.46
	W7. Looking woozy or clumsy in front of others	0.68
	W8. Losing control due to low BG	0.59
	W10. Making a mistake or having an accident at school	0.76
	W11. Getting in trouble at school due to low BG symptoms	0.72
	W12. Having seizures	0.53

**Table 4:** Pairwise correlations between the resulting PHFS factors.

	Maintain High BG	Helplessness/Worry about Low BG	Worry about Negative Social Consequences
Maintain High BG	─		
Helplessness/Worry About Low BG	0.196	─	
Worry About Negative Social Consequences	0.195	0.663	─

The analysis of the PHFS resulted in 3 factors, one for the Behavior and two for the Worry subscales. The Worry factors are strongly correlated with each other, while the correlations between any of the two Worry factors and the Behavior factor are weak.

**Table 5:** Pairwise correlations between the resulting CHFS factors.

	Maintain High BG	Avoid/Prevent Low BG	Helplessness/Worry about Low BG	Worry about Negative Social Consequences
Maintain High BG	─	0.402	0.144	0.089
Avoid/Prevent Low BG	0.402	─	0.169	0.134
Helplessness/Worry About Low BG	0.144	0.169	─	0.626
Worry About Negative Social Consequences	0.089	0.134	0.626	─

The analysis of the CHFS resulted in 4 factors, two for each subscale. The Worry factors are strongly correlated with each other, the Behavior factors are moderately correlated, and all other cross-correlations are weak.

**Table 6:** Pairwise correlations between factors of the HFS and the subscales of the PedsQL^TM^.

P-HFS	PEDs 1-11	PEDs 12-15	PEDs 16-22	PEDs 23-25	PEDs 26-28	PEDs Total
Maintain High BG	0.052	0.046	0.122	0.014	0.098	0.103
HFS Worry	0.314 **	0.123	0.199 *	0.269 **	0.201 *	0.333 **
Helplessness/Worry about Low BG	0.274 **	0.107	0.206 *	0.248 *	0.204 *	0.310 **
Worry about Negative Social Consequences	0.350 **	0.143	0.099	0.254 *	0.121	0.304 **
PHFS Total	0.311 **	0.127	0.204 *	0.256 *	0.211 *	0.337 **
HFS Behavior	0.190	0.136	0.054	0.057	0.099	0.161
Maintain High BG	0.158	0.177	0.177	0.116	0.196	0.224 *
Avoid/Prevent Low BG	0.147	0.064	−0.042	0.002	0.013	0.070
HFS Worry	0.360 **	0.327 **	0.324 **	0.401 **	0.097	0.421 **
Helplessness/Worry about Low BG	0.348 **	0.295 **	0.272 **	0.399 **	0.071	0.388 **
Worry about Negative Social Consequences	0.304 **	0.293 **	0.303 **	0.332 **	0.097	0.370 **
C-HFS Total	0.376 **	0.322 **	0.277 **	0.337 **	0.128	0.408 **

* Correlation is significant at the 0.05 level (2-tailed); ** correlation is significant at the 0.01 level (2-tailed).

## Data Availability

The data presented in this study are available upon reasonable request from the corresponding author.

## References

[1] Workgroup on Hypoglycemia, Americal Diabetes Association (2005). Defining and Reporting Hypoglycemia in Diabetes: A report from the American Diabetes Association Workgroup on Hypoglycemia. Diabetes Care.

[2] Seaquist E.R., Anderson J., Childs B., Cryer P., Dagogo-Jack S., Fish L., Heller S.R., Rodriguez H., Rosenzweig J., Vigersky R. (2013). Hypoglycemia and Diabetes: A Report of a Workgroup of the American Diabetes Association and The Endocrine Society. Diabetes Care.

[3] Kent D.A., Quinn L. (2018). Factors That Affect Quality of Life in Young Adults with Type 1 Diabetes. Diabetes Educ..

[4] Martyn-Nemeth P., Quinn L., Penckofer S., Park C., Hofer V., Burke L. (2017). Fear of hypoglycemia: Influence on glycemic variability and self-management behavior in young adults with type 1 diabetes. J. Diabetes Complicat..

[5] Barnard K., Thomas S., Royle P., Noyes K., Waugh N. (2010). Fear of hypoglycaemia in parents of young children with type 1 diabetes: A systematic review. BMC Pediatr..

[6] Gonder-Frederick L.A., Fisher C.D., Ritterband L.M., Cox D.J., Hou L., DasGupta A.A., Clarke W.L. (2006). Predictors of fear of hypoglycemia in adolescents with type 1 diabetes and their parents. Pediatr. Diabetes.

[7] Green L.B., Wysocki T., Reineck B.M. (1990). Fear of hypoglycemia in children and adolescents with diabetes. J. Pediatr. Psychol..

[8] Gonder-Frederick L., Nyer M., Shepard J.A., Vajda K., Clarke W. (2011). Assessing fear of hypoglycemia in children with Type 1 diabetes and their parents. Diabetes Manag..

[9] Shepard J.A., Vajda K., Nyer M., Clarke W., Gonder-Frederick L. (2014). Understanding the construct of fear of hypoglycemia in pediatric type 1 diabetes. J. Pediatr. Psychol..

[10] Haugstvedt A., Wentzel-Larsen T., Aarflot M., Rokne B., Graue M. (2015). Assessing fear of hypoglycemia in a population-based study among parents of children with type 1 diabetes–psychometric properties of the hypoglycemia fear survey–parent version. BMC Endocr. Disord..

[11] Tumini S., Fioretti E., Rossi I., Cipriano P., Franchini S., Guidone P.I., Petrosino M.I., Saggino A., Tommasi M., Picconi L. (2022). Fear of hypoglycemia in children with type 1 diabetes and their parents: Validation of the Italian version of the Hypoglycemia Fear Survey for Children and for Parents. Pediatr. Diabetes.

[12] Patton S.R., Noser A.E., Clements M.A., Dolan L.M., Powers S.W. (2017). Reexamining the Hypoglycemia Fear Survey for Parents of Young Children in a Sample of Children Using Insulin Pumps. Diabetes Technol. Ther..

[13] Wild D., Grove A., Martin M., Eremenco S., McElroy S., Verjee-Lorenz A., Erikson P., ISPOR Task Force for Translation and Cultural Adaptation (2005). Principles of Good Practice for the Translation and Cultural Adaptation Process for Patient-Reported Outcomes (PRO) Measures: Report of the ISPOR Task Force for Translation and Cultural Adaptation. Value Health.

[14] Tabachnick B.G., Fidell L.S. (2013). Using Multivariate Statistics.

[15] Field A. (2013). Discovering Statistics Using IBM SPSS Statistics.

[16] Kline P. (1993). Handbook of Psychological Testing.

[17] Varni J.W., Seid M., Rode C.A. (1999). The PedsQLTM: Measurement Model for the Pediatric Quality of Life Inventory. Med. Care.

[18] Johnson S.R., Cooper M.N., Davis E.A., Jones T.W. (2013). Hypoglycaemia, fear of hypoglycaemia and quality of life in children with Type 1 diabetes and their parents. Diabet Med..

